# Density Functional Theory‐Based Investigation of Adsorption, Permeation, and Desorption of Hydrogen Isotopes in Fe–Cr Alloy of Varied Compositions

**DOI:** 10.1002/cphc.70501

**Published:** 2026-07-26

**Authors:** Anil Boda, Sk. MusharafAli

**Affiliations:** ^1^ Chemical Engineering Division Bhabha Atomic Research Centre Mumbai India; ^2^ Homi Bhabha National Institute Mumbai India

**Keywords:** density functional theory (DFT), diffusion, Fe–Cr, H‐isotopes, permeation, solubility

## Abstract

A fundamental understanding of hydrogen behavior in Fe–Cr alloys is critical for mitigating hydrogen‐related challenges in fusion and fission reactor environments. Extensive calculations are performed using plane‐wave density functional theory to investigate the interaction and dynamics of hydrogen isotopes in a model bcc Fe–Cr system. The hydrogen dissociative adsorption on Fe–Cr surfaces is found to be exothermic, whereas absorption into subsurface and bulk sites is shown to be endothermic. The findings reveal that the tetrahedral‐site absorption energies for hydrogen become increasingly endothermic with rising Cr concentration. Nudged elastic band (NEB) calculations show that the predicted diffusion barriers of hydrogen isotopes increase with Cr content and thus reduce the permeation. The computed diffusion, permeation, and solubility trends agree with the experimental results. The analysis of hydrogen isotopes highlights the role of zero‐point energy in governing the desorption kinetics of hydrogen.

## Graphical abstract

The energy profile of H from tetrahedral to a nearby tetrahedral void is changed with changes in Cr content. The diffusion coefficients of H are also found to vary with variation in the Cr content in the Fe–Cr alloy. These changes in energy and diffusion are due to the altered electronic interaction of hydrogen within the alloy matrix with varying Cr content.

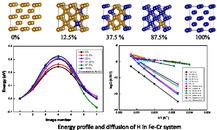

## 1 Introduction

A surge in global energy demand, along with issues such as carbon emissions and climate change, has compelled the international community to pursue the use of renewable energy sources rather than conventional fossil fuels to procure green energy. In this regard, the International Thermonuclear Experimental Reactor (ITER) is an aspiring energy project that primarily focuses on the study of nuclear fusion reactions and providing a major impetus for fusion power plants. ITER emphasizes on exhibiting the control of fusion reactions and plasma with negligible ramifications to the environment. Heavier isotopes of hydrogen, such as deuterium and tritium are touted as the fuel for the first‐generation fusion reactors. The transmutation effect, along with tritium creation using a lithium blanket, forms a significant amount of H and its isotopes in the reactor. Tritium, being radioactive in nature, along with its ability to readily exchange with hydrogen poses a major concern of radiation hazard if exposed to living creatures. This has led to numerous computational and experimental studies to identify materials that can resist extreme heat and particle fluxes during the nuclear fusion process.

Metal alloys are considered to be the most suitable as structural materials for the containment of hydrogen and its radioactive isotopes. However, the interaction of such alloys with hydrogen at an atomistic level has a negative effect on their mechanical and physical properties. Hydrogen tends to accumulate in the microstructural defects of the alloy, leading to its degradation. This phenomenon is known as hydrogen embrittlement [1, 2, 3]. It is imperative to conduct an atomistic level study of diffusivity for hydrogen isotopes in metallic bulk systems that will be utilized further in the prediction of their solubility and permeability. The use of steel has garnered significant attention as a structural material [4, 5] and a plasma coating substance [6, 7, 8]. Ferritic–martensitic stainless steels in particular have received renewed interest in the past few years [9, 10, 11, 12]. These steels exhibit a bcc structure with a high chromium content ranging from 2% to 20%. These alloys are low cost, radiation resistant, and display favorable properties such as a high melting point and good ductility. The presence of high chromium content enhances their corrosion resistance. It is also observed that steady state permeation coefficients of hydrogen for steels having 9%–13% chromium are lower by one order of magnitude than those of pure Fe. These iron–chromium steel alloys may be utilized as structural materials for light waterreactors [13, 14], nuclear fusion reactors [15, 16], solid oxide fuel cell applications [17, 18, 19, 20], and Generation IV reactors [21, 22]. Understanding the behavior hydrogen isotopes, especially their radioactive isotope, in pure metal and metallic alloy is extremely difficult and they are very scarce and also the results are widely varied. The bcc Fe–Cr binary alloy serves as a model system for studying ferritic steels and binary alloy systems [23, 24, 25]. The effect of hydrogen on Fe–Cr alloys has been extensively studied to understand its influence on surface and bulk properties. Gupta et al. [23] investigated, using first‐principles calculations, how surface hydrogen modifies the anomalous surface segregation behavior of Cr in Fe‐rich Fe–Cr alloys, revealing that hydrogen significantly alters the segregation tendencies of Cr atoms. Peñalva et al. [24] experimentally analyzed the role of Cr concentration on hydrogen iffusion and trapping in Fe–Cr alloys, demonstrating that increasing Cr content influences both the diffusion coefficients and trapping characteristics of hydrogen. Complementing these findings, Samin et al. [25] employed first‐principles localized cluster expansion methods to study hydrogen diffusion kinetics in homogeneous and heterogeneous Fe–Cr alloys, providing atomistic insights into hydrogen transport mechanisms and their dependence on alloy composition and local atomic environments. Further, density functional theory (DFT) has been used to study the interaction of H with the Fe–Cr system, in particular Fe15Cr [26]. By and large, hydrogen diffusion in solids was calculated by considering the total electronic energy of the relaxed structures in the ground state and transition state as a reference with zero‐point energy corrections as reported in our previous studies [27, 28, 29, 30]. Most of the studies are for pure metal and few are for binary and ternary alloy. To the best of our knowledge, a fundamental study using DFT is yet to be reported on the permeation of H isotopes in the Fe–Cr binary system. This motivates us to investigate the permeation of hydrogen isotopes in the Fe–Cr system. The work presented herein focuses on the study of adsorption, diffusion, permeation, solubility, and desorption of hydrogen isotopes in the Fe–Cr system using first principles DFT calculations.

The present work is organized as follows. Section 2 describes the computational protocol based on first‐principles DFT employed to model hydrogen isotope interactions with surface and bulk Fe–Cr systems. In Section 3.1, the dissociative adsorption behavior of H isotopes on the Fe–Cr(100) surface with 12.5% Cr is investigated. Section 3.2 examines multiple hydrogen adsorption configurations on the Fe–Cr(100) surface to assess coverage‐dependent effects. Hydrogen absorption at interstitial sites in bulk Fe–Cr is discussed in Section 3.3, followed by an analysis of hydrogen isotope diffusion pathways and diffusion barriers in bulk Fe–Cr in Sections 3.4 and 3.5 focuses on hydrogen permeation and solubility characteristics derived from absorption and diffusion energetics. The influence of isotope effects on diffusion and permeation is systematically analyzed in Section 3.6. Thermal desorption spectra of hydrogen from the Fe–Cr(100) surface containing 12.5% Cr are presented in Section 3.7 to elucidate desorption behavior. Finally, the main conclusions of the study are summarized in Section 4.

## 2 Computational Methods

The simulations in the present study were carried out using DFT [31, 32, 33] based on the Vienna Ab‐initio Simulation Package (VASP) [34, 35]. The generalized gradient approximation‐based Perdew−Burke−Ernzerhof functional [36, 37] was used for electron exchange and correlation. The ionic cores were represented by the projector augmented wave (PAW) potential with spin polarization and an energy cutoff of 400 eV. Integration in the Brillouin zone was carried out by Gamma‐centered K‐points [38].

For the bulk Fe–Cr system containing H atoms, k‐point samplings of 8 × 8 × 8 for Fe_15_Cr_1_ and Fe_14_Cr_2_were used. During the calculations, the atomic positions as well as the supercell size were relaxed to equilibrium. The forces on all the atoms are kept less than 0.01 eV Å^−1^. The zero‐point energy (=1/2i *hv*_*i*_, *v*_*i*_ is the frequency) was determined from the H atom frequency by performing phonon calculations by freezing the Fe and Cr atoms. The contribution of metal atoms to the total ZPE corrections can be safely ignored compared to lighter H isotopes due to their lower mass, as reported earlier. A k‐point sampling of 8 × 8 × 8 for bulk FeCr16 was used, whereas a 2 × 2 surface cell with eight layers of metal and a k‐point sampling of 6 × 6 × 1 was followed for the H atom at the surface of FeCr(100). Furthermore, 10 Å of vacuum was provided for the calculations of the surface. The nudged elastic band (NEB) method [39] was performed to find the minimum energy paths and the transition states for H atom diffusion into FeCr through FeCr(100) [using the surface (2 × 2 × 2) cell] and bulk FeCr. The convergence criterion was maintained by setting the force of about 0.01 eV  Å^−1^ or less acting on an atom for all the images.

## 3 Results and Discussion

### 3.1 Model System

As an initial step toward investigating 9%–12% Cr ferritic–martensitic steels [23, 24, 25], we have selected the Fe_14_Cr_2_ alloy (Fe–12.5 at.% Cr) modeled using a 2 × 2 × 2 bcc unit cell, which falls within the experimentally relevant compositional range. Owing to the relatively small number of atoms, this system is well‐suited for ab initio studies. Using this composition, we examine hydrogen dissociation, adsorption, absorption, diffusion, permeation, solubility, and thermal desorption. In addition, the absorption, diffusion, and permeation behavior of hydrogen isotopes are investigated as a function of Cr concentration over a wide range (0%–100% Cr) (Figure 4), which necessitates a large number of calculations. The use of a reduced supercell significantly lowers the computational cost while enabling systematic compositional variation.

### 3.2 Dissociative Adsorption of H_2_ Isotopes on FeCr (100) (87.5%:12.5%) Surface

**FIGURE 1:**
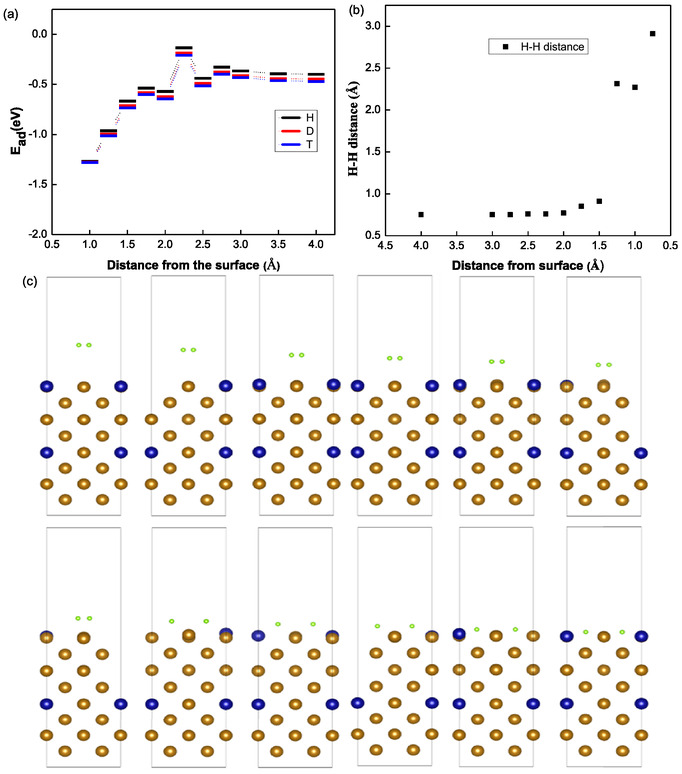
(a) Potential energy surface for dissociative adsorption of H_2_ molecule on FeCr(100) surface against the relative distance of H_2_ molecule from the surface (b) variations of the H—H bond distances. (c) Visuals of dissociation route of H_2_ molecule from bridge site to hollow site at FeCr(100) (87.5%:12.5%) surface. Green spheres represent H, brown spheres represent Fe, and blue spheres represent Cr atom.

A dissociative chemisorption of H_2_ isotopes can readily take place on the FeCr surface (Fe–12.5 at.% Cr) and the corresponding pathway followed by H_2_ isotopes. In the present calculations, we have considered the bridge position as the starting adsorption sites in which the H_2_ molecule is placed parallel to the surface. In the calculations, H_2_ molecule was sequentially moved toward the surface and the positions of the atoms were allowed to relax parallel to the surface plane. It was observed that dissociation of the H_2_ molecule takes place in close proximity to the surface. The results related to adsorption at the top and bridge sites are depicted in Figure 1. The H_2_ molecule dissociates as it approaches the surface. After dissociation, two adsorbed H atoms occupy hollow sites. Initially, the molecular adsorption of H_2_ molecule takes place and the adsorption energies are in the range of 40–80 meV, which indicates that the adsorption is physisorption. From the figure, it can be seen that, first, the molecule approaches to a distance of 1.5 Å from the surface, followed by the molecular dissociation and finally to two chemisorbed H atoms at hollow site.

### 3.3 Multiple Adsorptions of H Atoms on FeCr (100) Surface

**FIGURE 2:**
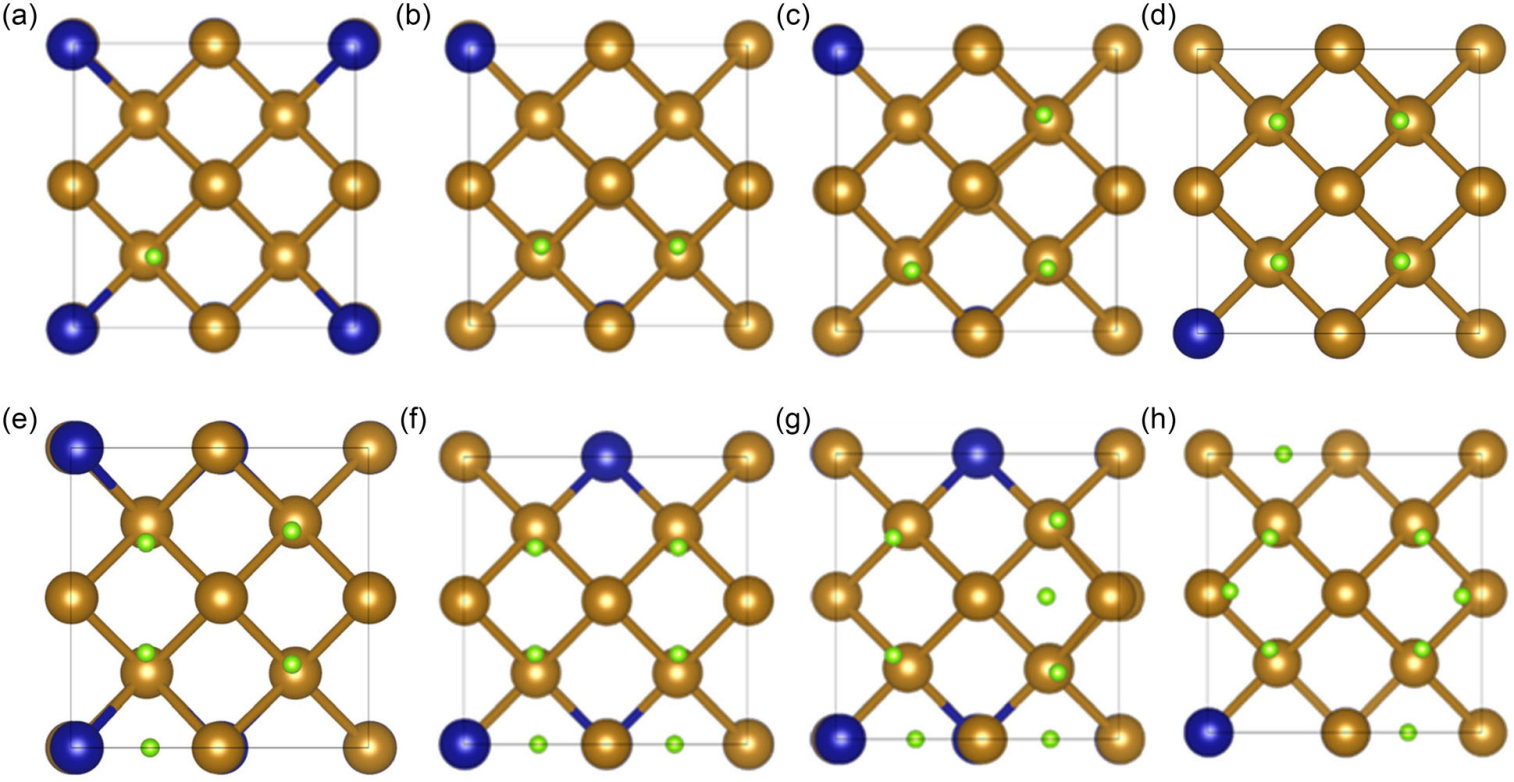
Representative structures of optimized surface of FeCr(100) (87.5%:12.5%) with different coverage of H atom (a) 0.25 ML, (b) 0.5 ML, (c) 0.75 ML, (d) 1.0 ML, (e) 1.25 ML, (f) 1.50 ML, (g) 1.75 ML, and (h) 2.0 ML.

In this section, the result of adsorption of multiple H atoms on 0% Cr (pure Fe) and 100% Cr surfaces and FeCr (100) surface (12.5% Cr) are presented. The optimized structures of H atom containing FeCr (100) surface from 0.25 to 2.0 ML coverage (one H atom on the surface) are depicted in Figure 2. The adsorption energy (*E*_ad_) of the H atom on the FeCr surface was determined using the relation given below:

(1) $$E_{\mathrm{ad}}=E_{\mathrm{nH}-\mathrm{FeCr}(\mathrm{slab})}-E_{\mathrm{FeCr}(\mathrm{slab})}\;-\;n\frac{1}{2}E_{H_{2}}$$

Here, $E_{H_{2}}$, *E*_FeCr(slab)_ and *E*_*n*H−FeCr(slab)_, represent the energy of a free H_2_ molecule, FeCr (100) slab and H‐containing Fe (100) slab, respectively.

**FIGURE 3:**
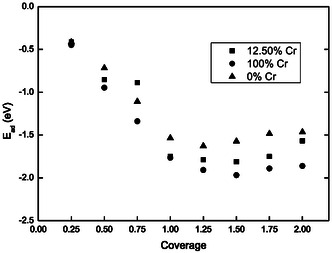
Adsorption energy of H on 0% Cr (pure Fe (100)), 100% Cr (pure Cr (100)), and FeCr (100) (87.5%: 12.5%) surfaces with variation in the H‐coverage.

The adsorption of multiple H atoms on the Fe (100), FeCr (100), and Cr (100) surfaces was investigated to understand the effect of hydrogen coverage and Cr concentration on the adsorption behavior, as shown in Figure 3. For all three surfaces, the adsorption energy becomes increasingly negative with increasing H coverage up to 1.0 ML, indicating stronger H adsorption at low coverages. Beyond 1.0 ML, the adsorption energies gradually approach saturation up to 2.0 ML, which may be attributed to the reduced metal–H interaction strength and increased H–H interactions at higher H coverages [28, 30, 40, 41]. Among the three systems, the adsorption energies on the pure Cr surface are the most negative, demonstrating that hydrogen adsorption is energetically more favorable on Cr compared to pure Fe. The FeCr (100) surface containing 87.5% Fe and 12.5% Cr exhibits intermediate behavior between the two limiting cases. These results suggest that while Cr weakens H stability in the bulk Fe–Cr alloy, it enhances H binding at the surface. The contrasting behavior between surface adsorption and bulk absorption originates from the differences in the local electronic environments at the surface and in the bulk. The number of possible hydrogen arrangements increases significantly with increasing H coverage. Therefore, instead of exhaustively considering all possible configurations, a systematic sequential adsorption approach was adopted to capture the overall adsorption trends. Hydrogen atoms were added progressively to the most energetically favorable available adsorption sites. Stable hollow‐site adsorption configurations were first considered up to 1.0 ML coverage, followed by bridge‐site adsorption configurations up to 2.0 ML coverage [28]. This methodology provides a physically meaningful description of progressive hydrogen accumulation on the surface while maintaining computational feasibility. Since the primary objective of this work is to understand the overall trends in hydrogen adsorption behavior with Cr concentration and H coverage, the adopted approach is sufficient to establish the comparative adsorption characteristics of Fe, Fe–Cr, and Cr surfaces.

### 3.4 Absorption of H Atoms in Bulk FeCr

The absorption energy (*E*_ab_) of H atom on FeCr surface was determined using the relation given below:

(2) $$E_{\mathrm{ab}}=E_{\mathrm{nH}-\mathrm{FeCr}(\mathrm{bulk})}-E_{\mathrm{FeCr}(\mathrm{bulk})}\;-\;n\frac{1}{2}E_{H_{2}}$$

Here, $E_{H_{2}}$, *E*_FeCr(bulk)_ and *E*_nH−FeCr(bulk)_, represent the energy of free H_2_ molecule, FeCr bulk and H containing bulk FeCr, respectively. The calculated absorption energy at different site is listed in Table 1.

**TABLE 1:** Calculated values of absorption energies (*E*_ab_) for hydrogen atoms in bulk FeCr.

Bulk structures	*E*_ab_, eV	Δ*E *= *E*_o_ − *E*_t_, eV
Fe_15_Cr‐H_o_	0.300	0.055
Fe_15_Cr‐H_t_	0.245	
Fe_14_Cr_2_‐H_o_	0.477	0.125
Fe_14_Cr_2_‐H_t_	0.352	

The calculated Fe—Fe bond lengths are 2.833 and 2.453 Å, with the lattice constant being 2.833 Å, while the experimental lattice constant [42] is 2.87 Å. Similarly, the Cr—Cr bond lengths are 2.839 and 2.458 Å, with the calculated lattice constant [42] of 2.839 Å and the experimental value of 2.91 Å. The Fe—Cr bond length is 2.458 Å, with a lattice constant of 2.835 Å, while the experimental lattice constant [43] for Fe–13.62%Cr is 2.872 Å. For hydrogen interactions, the Fe—H bond length is 1.571 Å, compared to 1.583 Å in pure Fe, and the Cr—H bond length is 1.641 Å, compared to 1.586 Å in pure Cr. The slight decrease in Fe—H bond length and the increase in Cr—H bond length indicate differing hydrogen interactions in Fe and Cr environments, possibly due to charge redistribution. Further, from absorption energy values presented in Table 1, H‐absorption in the tetrahedral void was found to be more stable compared to nearby octahedral void absorption.

**FIGURE 4:**
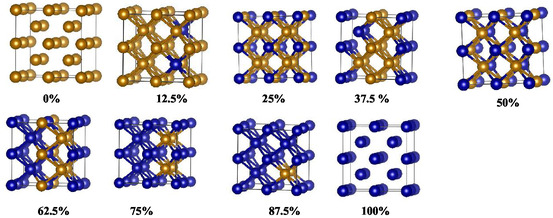
Computed relaxed structure of Fe–Cr system with variation in the Cr‐content.

**FIGURE 5:**
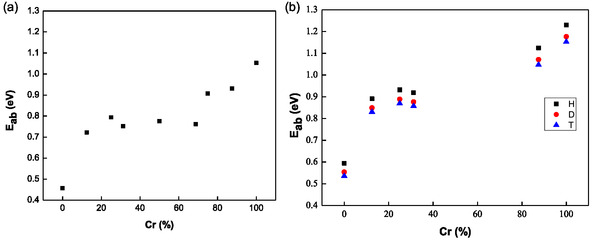
Plot of variation in the absorption energy of (a) H and (b) H isotopes in the tetrahedral void of bulk Fe–Cr with variable Cr content.

Further, to study the effect of Cr‐content on the absorption of H, systematically varied the concentration of Cr in bulk Fe. The relaxed structures are presented in Figure 4. The absorption energy is calculated for H in a tetrahedral void with variation in the Cr content of bulk Fe. An increase in the endothermicity is obtained with an increase in the Cr content. The calculated values are displayed in Figure 5. Further, the computed values of zero‐point energy corrected absorption energy are presented in Figure 5. The ZPE corrected adsorption energy of the T atom was seen to be greater than that of H/D isotopes with an increase in the Cr‐content in bulk Fe. An increase in Cr content leads to higher absorption energy, indicating increasing endothermicity. Consequently, this results in reduced solubility. The solubility trend follows: *T* > *D* > *H*.

**FIGURE 6:**
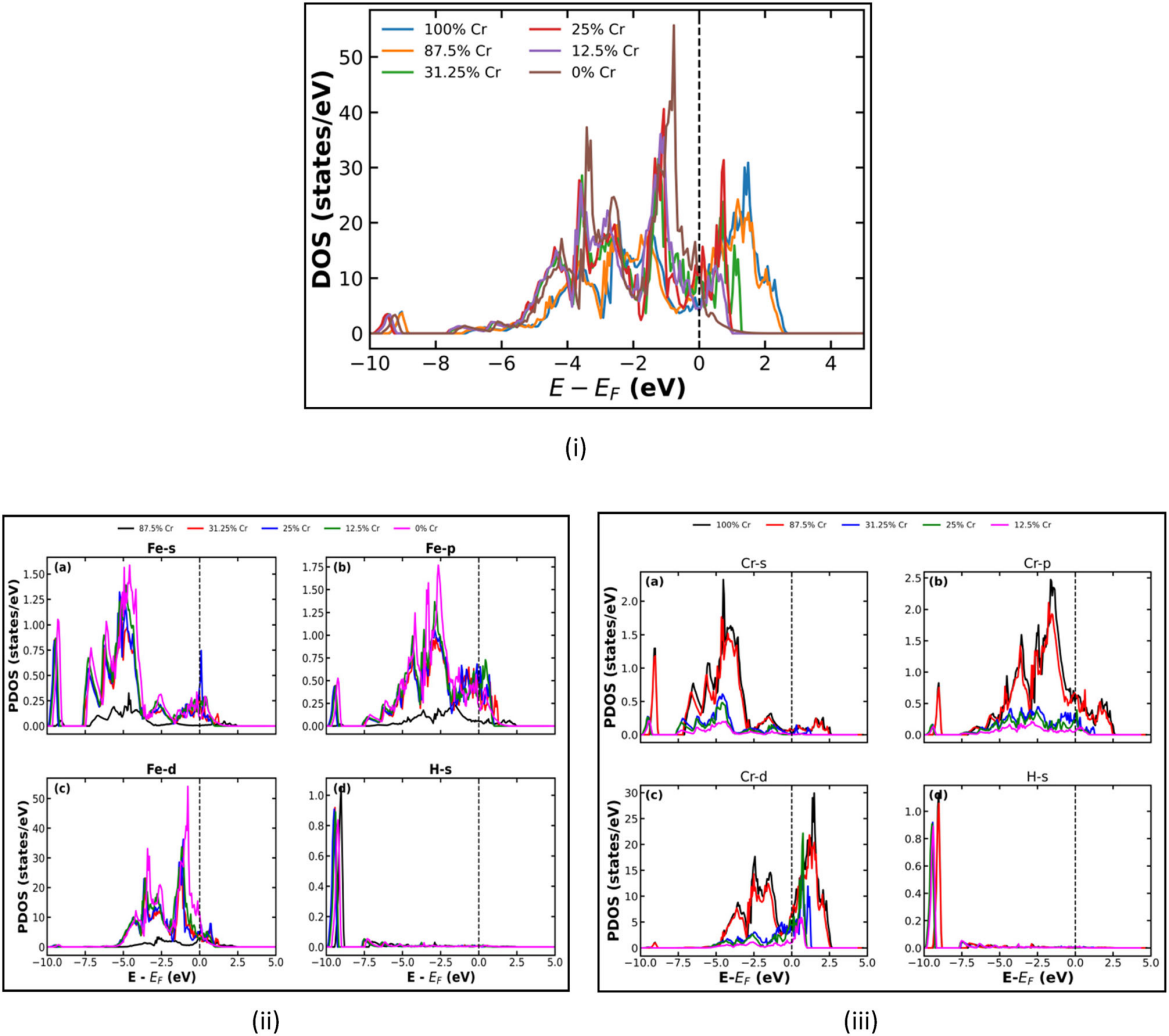
Plot of calculated (i) total density of states, TDOS and (ii) projected density of states, PDOS of Fe and H (iii) PDOS of Cr and H with variation of Cr content in *H*_t_/bulk‐Fe–Cr system.

**FIGURE 7:**
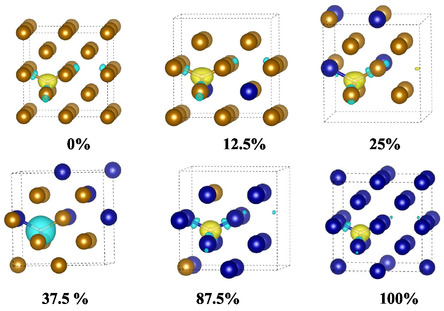
Calculated values of differential charge densities with variation of Cr content in *H*_t_/bulk‐Fe–Cr system. isosurface was set to ± 0.004 e Å^−3^.

Furthermore, the kind of H atom absorption in bulk Fe–Cr is also studied by calculating the PDOS, as shown in Figure 6. The absorption of H in bulk Fe–Cr is governed by its interaction with Fe and Cr orbitals. H(1*s*) interacts strongly with both Fe and Cr 4s and 4p orbitals, with a stronger interaction observed in Fe compared to Cr. As the Cr content increases in the bulk, a significant shift in H(1*s*) energy toward higher values is observed, indicating a weakening of interaction between H and the surrounding metal atoms. This suggests that H absorption becomes less favorable with increasing Cr content due to a reduction in bonding strength. The observed shift in energy aligns with the trend of decreasing absorption capacity in Fe–Cr alloys with higher Cr concentrations, as the presence of Cr reduces the ability of the bulk material to effectively retain H atoms (Figure 7).

### 3.5 Diffusion of H Isotopes Through Bulk FeCr

It is important to study the diffusion behavior of H with varying concentration of Cr in the FeCr system.

The common expression for the diffusion of interstitial atoms in the solid is

(3) $$D=D_{0}\exp(-E_{a}/kT)$$

Where, *D*_0_ is typically the temperature independent pre‐exponential factor, *kT* is the Boltzmann constant time temperature. Here, *E*_a_ represents the activation barrier which has to be surmounted by the interstitial atom from an equilibrium location to move forward along the diffusion path to another equilibrium position. The rate of diffusion of an atom using the theory of Wert and Zener [44] can be expressed as:

(4) $$D=\frac{1}{6}l^{2}\Gamma$$

Where “*l*” is the jump length from one interstitial site to another interstitial site and Γ represents the jump rate, which can be calculated using the activated complex model of Eyring [] within the transition state formula by Wigner [45, 46] as:

(5) $$\Gamma =\frac{kT}{h}\times \frac{Q^{\#}}{Q}\exp^{\frac{-\text{Δ}E}{kT}}$$

Here, *Q* ^#^ and *Q* represent the total partition function of activated and stable sites of the molecular complex. Δ *E* denotes the energy difference between the activated and stable sites. The vibrational partition function under the classical mechanics solution leads to Vineyard's [47] result, which can be expressed as:

(6) $$\Gamma_{\mathrm{cl}}=\frac{\prod_{j=1}^{3N}\text{ϑ}j}{\prod_{j=1}^{3N-1}{\text{ϑ}}^{\#}j}\exp^{\frac{-[\text{Δ}\text{Ea}+\text{Δ}\text{ZPE}]}{kT}}$$

here, *ϑ j* and *ϑ* ^#^ *j* denote the frequency of the ground and transition state and the jump rate can be written as

(7) $$\Gamma_{\mathrm{hTST}}=\frac{kT}{h}\times \frac{\prod_{j=1}^{3N}\frac{e^{\frac{-h\text{ϑ}j^{\#}}{2kT}}}{1-e^{\frac{-h\text{ϑ}j^{\#}}{kT}}}}{\prod_{j=1}^{3N-1}\frac{e^{\frac{-h\text{ϑ}j}{2kT}}}{1-e^{\frac{-h\text{ϑ}j}{kT}}}}\exp^{\frac{-[\text{Δ}\text{Ea}+\text{Δ}\text{ZPE}]}{kT}}$$

### 3.6 Permeation and Solubility

The value of permeability for a gas can be used to gain insight into the permeation characteristics and subsequently for screening and designing of novel barrier materials. The permeability (*Q*) of a gas can be calculated by using Sieverts’ constant, *K*_s_(*T*) and diffusivity, *D*, as per the below relation [48]:

(8) $$\Theta =\frac{1}{2}K_{s}(T)D$$

The Sieverts’ law can be applied for the determination of the solubility of dilute hydrogen gas. The *K*_s_(*T*) is evaluated by the expression given below:

(9) $$K_{s}(T)=\exp(\beta [\sum_{i}\frac{{hw}_{i}}{4}‐E_{b}‐\sum_{i}\frac{h\vartheta_{i}}{4}])\frac{1}{\sqrt{\alpha }}\{\sqrt{1-\exp[-\text{β}\sum_{i}\frac{hw_{i}}{2}]}\}\frac{1}{\prod_{i}(1-\exp(-\text{β}(h{\text{ϑ}}_{i})))}$$

$$\alpha ={\left[\frac{2\Pi mkT}{h^{2}}\right]}^{\frac{3}{2}}\left\{\frac{4\Pi^{2}I{\left(kT\right)}^{2}}{h^{2}}\right\}$$

$$\beta =\frac{1}{kT}$$

where *ϑi* is the vibrational frequency of a free hydrogen molecule. *w*_*i*_ is the vibrational frequency of the H atom in bulk FeCr. *E*_b_ is the binding energy of the H atom with bulk FeCr. “*m*” and “*I*” represent the mass and moment of inertia of the H_2_ molecule. *k*, *T*, and *h* are the Boltzmann constant, temperature, and Planck's constant, respectively.

**FIGURE 8:**
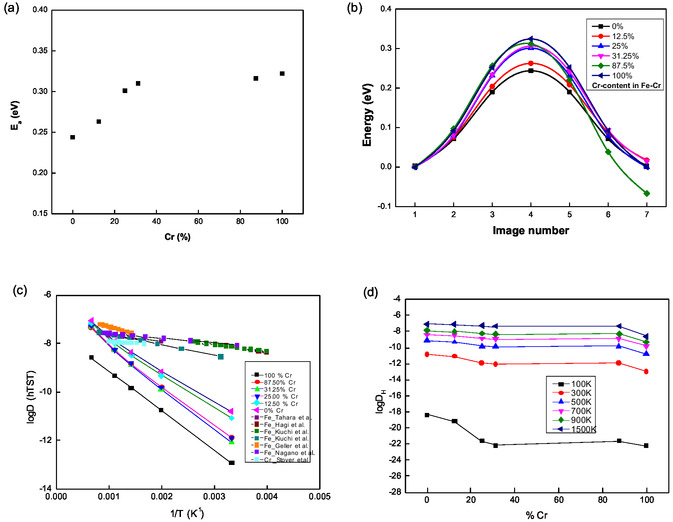
Plot of the variation in the (a) activation energy and (b) energy profile of H from tetrahedral void to nearby tetrahedral void in bulk Fe–Cr with variable Cr content. (c,d) Plot of calculated diffusion coefficients (m^2^ s^−1^) of H at different temperatures with variation in the Cr content in Fe–Cr system.Tahara et al. [49], Hagi et al. [50, 51], Kiuchi et al. [52], Geller et al. [53], Nagano et al. [54], and Stover et al. [55].

NEB calculations were conducted for the diffusion of an H atom from a tetrahedral to a nearby tetrahedral with variation in the Cr content of bulk Fe. The activation barrier is found to be increased with an increase in the Cr content. The results are displayed in Figure 8. The increase in the diffusion activation barrier with increasing Cr concentration in Fe–Cr alloys can be understood from the changes in the electronic interaction of hydrogen within the alloy matrix. As the Cr content increases, the absorption energy of H in bulk Fe–Cr becomes progressively more endothermic, indicating that hydrogen becomes less energetically favorable in the alloy environment. This reduced stability of H in the interstitial sites increases the energy required for H migration, thereby leading to a higher diffusion activation barrier.

The electronic structure analysis further supports this behavior. The density of states (DOS) shows a stronger orbital overlap and hybridization between H and Fe atoms in pure Fe compared to that between H and Cr in pure Cr. The stronger Fe–H interaction stabilizes hydrogen in Fe‐rich environments and facilitates easier hopping between neighboring interstitial sites, resulting in lower diffusion barriers. In contrast, the weaker H–Cr interaction reduces the degree of electronic hybridization and bonding stabilization around hydrogen. This may be the reason for the decrease in the permeation of H with an increase in the Cr content.

The calculated values of diffusion coefficient values, along with experimental results, are presented in Figure 8. The diffusion values of hydrogen (H) and its isotope deuterium (D) increase with rising temperature, as expected due to the enhanced atomic mobility at higher temperatures. This trend is consistent with experimental data for pure iron (Fe) and chromium (Cr), where the diffusion of hydrogen and its isotope is more pronounced at elevated temperatures. In the Fe–Cr system, the diffusion values are found to be between those of pure Fe and pure Cr, indicating that the diffusion characteristics of hydrogen in the alloy are influenced by its composition. Experimental observations further reveal that as the chromium content increases, the diffusion values decrease. This reduction in diffusion is attributed to the influence of chromium atoms. Consequently, higher chromium content in the Fe–Cr system leads to slower diffusion rates for hydrogen and its isotopes.

**FIGURE 9:**
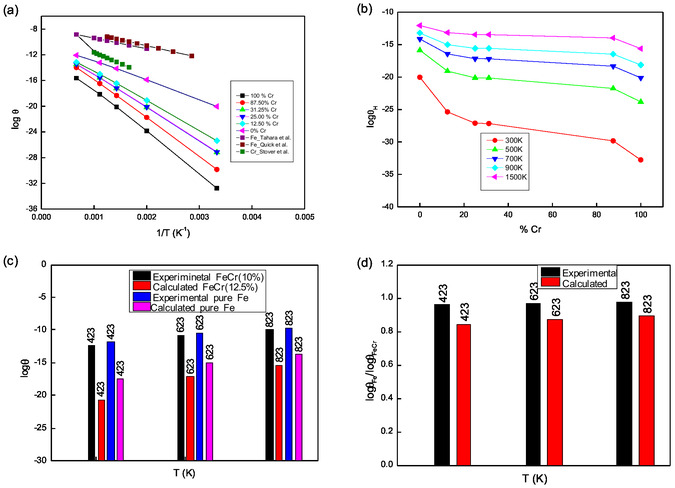
Plot of calculated permeability coefficients (*θ*, mol m^−^^1^ s^−^^1^ Pa^−0.5^) of H at different temperatures with variation in the Cr content in the Fe–Cr system. Tahara et al. [49], Quick et al. [56], and Stover et al. [55]. (c,d) Plot of calculated and experimental permeability coefficients (*θ*, mol m^−^^1^ s^−^^1^ Pa^−0.5^) of H at different temperatures Exp [57].

The calculated values of permeability coefficients along with experimental results are presented in Figure 9. The permeation values of hydrogen (H) and its isotope deuterium (D) are observed to increase with rising temperature, consistent with the behavior seen in diffusion. Higher temperatures enhance atomic mobility, making it easier for hydrogen to permeate through materials. This trend aligns with experimental observations, where increased temperature facilitates the movement of hydrogen atoms through the material. In the Fe–Cr system, the permeation values are found to decrease with increasing chromium content, similar to the trend observed for diffusion.

**FIGURE 10:**
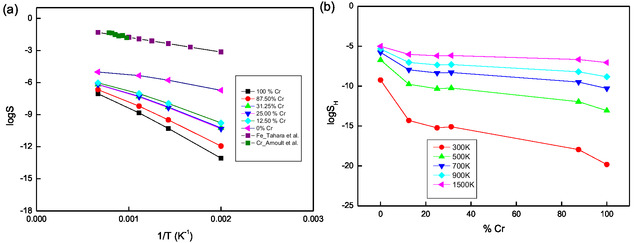
Plot of calculated solubility (*S* mol m^−^^1^ Pa^−0.5^) of H at different temperatures with variation in the Cr content in Fe–Cr system. Tahara et al. [49], Arnoult et al. [58].

The calculated values of solubility, along with experimental results, are presented in Figure 10. The solubility values of hydrogen (H) and its isotope deuterium (D) exhibit a clear temperature dependence, with solubility increasing as the temperature rises. This behavior is in line with experimental observations, as higher temperatures facilitate greater dissolution of hydrogen into the material. In the Fe–Cr system, the solubility values decrease with increasing chromium content, mirroring the trend observed for diffusion. This can be attributed to the fact that chromium forms a less favorable environment for hydrogen, reducing its ability to dissolve into the material. Experimental data confirms this, showing that as the chromium content in the alloy increases, the solubility of hydrogen decreases. Furthermore, solubility is higher in iron (Fe) compared to chromium (Cr), which is linked to the solution energy of hydrogen. The solution energy of hydrogen in chromium is more endothermic than in iron, meaning that it requires more energy for hydrogen to dissolve in chromium, making hydrogen solubility lower in Cr than in Fe.

### 3.7 Effect of Isotopes

**FIGURE 11:**
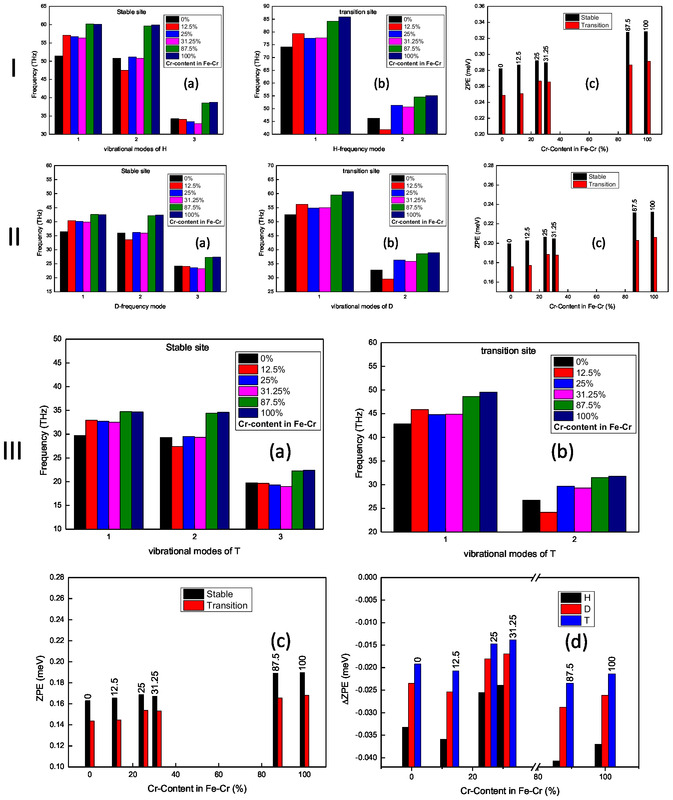
(I) Calculated values of H‐frequencies at stable and transition site and ZPE with variation of Cr‐content in Fe–Cr alloy. (II) Calculated values of D‐frequencies at stable and transition site and ZPE with variation of Cr‐content in Fe–Cr alloy. (III) Calculated values of T‐frequencies at stable and transition site and ZPE with variation of Cr‐content in Fe–Cr alloy. ZPE difference (ZPE_TS _− ZPE_stable_) with variation of Cr‐content in Fe–Cr alloy for H, D, and T.

The calculated values of H, D, and T‐frequencies at stable and transition site and ZPE with variation of Cr‐content in Fe–Cr alloy are presented in Figure 11. The zero‐point energy (ZPE) of a stable site is higher than that of the transition state, leading to a correction factor given by ΔZPE = ZPE(TS) − ZPE(S).

**FIGURE 12:**
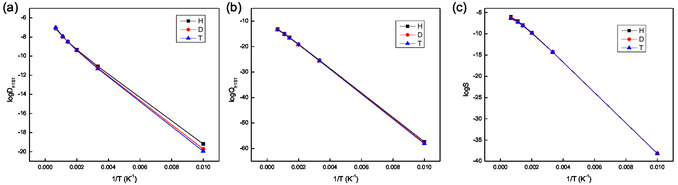
Calculated values of diffusion coefficients (m^2^ s^−1^), permeability coefficients (*Q*, mol m^−^^1^ s^−^^1^ Pa^−0.5^), and solubility (*S* mol m^−^^1^ Pa^−0.5^) of H, D, and T at different temperatures in Fe–Cr system (12.5% Cr).

Since this correction is negative, it reduces the diffusion barrier, modifying the activation energy as Ea′ = Ea + ΔZPE. The ZPE correction for hydrogen (H) is higher compared to that for deuterium (D) and tritium (T), which further reduces the barrier height for H diffusion, making it easier for H to migrate compared to its heavier isotopes. The effect of isotopes on the diffusion, permeation, and solubility of H isotopes in the Fe–Cr system was studied and displayed in Figure 12. As expected, the diffusion coefficient follows the trend H > D > T, consistent with the fact that lighter isotopes diffuse faster due to their lower mass. Similarly, permeability also follows the same order, with H exhibiting the highest permeability, followed by D and T, in agreement with theoretical and experimental findings [24, 27, 28, 30, 49, 55, 56]. Furthermore, increasing temperature enhances both diffusion and permeation for all isotopes, with the effect being more pronounced for the heavier isotopes D and T, as thermal energy helps overcome their relatively higher diffusion barriers.

### 3.8 Thermal Desorption Spectrum (TDS) of Hydrogen on the FeCr (100) Surface (12.5% Cr)

The dissociative adsorption energy of hydrogen on FeCr(100) is known to be coverage dependent (*θ*). DFT was employed to calculate the coverage dependence of the adsorption energy of H on the FeCr surface. Furthermore, the thermal desorption spectrum of hydrogen on the FeCr surface was simulated using DFT simulations and the Polanyi−Wigner equation to understand the surface desorption kinetics. The Polanyi−Wigner equation has been solved numerically using the Runge–Kutta method [59].

The rate of desorption is represented by

(10) $$r=-\frac{d\theta }{\mathrm{dt}}=\vartheta \left(\theta \right).\theta^{n}.e^{\frac{E_{d}}{kT}}$$

where *r* is the rate of desorption, *θ* is the coverage, *v* is the prefactor, *E*_d_ is the activation energy of desorption [60], and *n* is the desorption order. *θ* = 0.50 ML, *v* = 10^13^ s^−1^, and dt = 10 K were used for evaluating the rate of desorption. The Polanyi‐Wigner equation assumes a single desorption path with a well‐defined energy, but this is not accurate for activated adsorption/desorption with variable coverage. As a result, the equation can only provide an average value for desorption at a given temperature.

**FIGURE 13:**
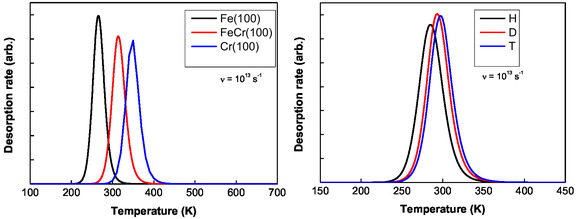
Calculate thermal desorption spectra of H_2_ on the Fe, Cr, and FeCr(100) surface (12.5% Cr) and TDS of H_2_, D_2_, and T_2_ on the FeCr(100) surface (12.5% Cr).

Calculated thermal desorption spectra of H_2_ on the Fe, Cr, and FeCr(100) surfaces are displayed in Figure 13. The desorption behavior of hydrogen (H_2_) varies significantly between Fe, FeCr, and pure Cr surfaces, with desorption occurring at lower temperatures for Fe compared to FeCr and Cr. This trend is attributed to the desorption energy barriers, where Fe has the lowest barrier at 0.718 eV, leading to hydrogen desorption beginning at a lower temperature. In contrast, the FeCr surface has a slightly higher desorption barrier of 0.854 eV, while pure Cr exhibits the highest barrier at 0.948 eV. Consequently, the desorption temperature peaks for these surfaces are observed at different temperatures, with Fe peaking at 265 K, FeCr at 310 K, and Cr at 350 K. The increasing desorption barriers from Fe to Cr imply stronger hydrogen binding on Cr surfaces, resulting in higher desorption temperatures and suggesting that FeCr alloys may offer a balanced hydrogen interaction.

The desorption of hydrogen isotopes (H_2_, D_2_, and T_2_) occurs at different temperatures, with H_2_ desorbing at a lower temperature compared to D_2_ and T_2_. This trend arises due to the stronger adsorption of heavier isotopes on the Fe–Cr surface, leading to higher desorption barriers. Specifically, the desorption barrier for H_2_ on Fe–Cr is 0.773 eV, which is lower than that of D_2_ (0.797 eV) and T_2_ (0.807 eV). As a result, the desorption peaks shift accordingly, with H desorbing at 285 K, D at 295 K, and T at 300 K. This isotope effect highlights the influence of mass‐dependent interactions on adsorption strength and desorption kinetics.

## 4 Conclusions

Iron and chromium are important constituents widely used in steel. Steels containing 9–12 wt% Cr have recently gained attention for potential applications as structural materials in nuclear reactors and prospective fusion reactors. These steels exhibit diffusion coefficients approximately one order of magnitude lower than those of pure iron. Computational modeling plays a vital role in addressing hydrogen behavior in Fe–Cr materials used in fusion reactors. This study utilizes DFT to investigate the interactions and dynamics of hydrogen isotopes within a bcc Fe–Cr lattice. NEB methods reveal that higher Cr content increases activation energy barriers, resulting in reduced hydrogen diffusion and permeation. Phonon calculations highlight isotope effects, showing that lighter isotopes diffuse and permeate more readily than heavier ones. The desorption behavior of hydrogen varies across Fe, FeCr, and Cr surfaces, with Fe exhibiting the lowest desorption barrier and temperature. Hydrogen isotope desorption follows a mass‐dependent trend, with heavier isotopes desorbing at higher temperatures due to stronger adsorption. These computational findings complement the experimental observations and provide critical insights into hydrogen‐related challenges, where experimental results are not available and thus might aid in the selection of right materials for fusion reactor technology.

## Conflicts of Interest

The authors declare no conflicts of interest.

## Supporting information

Supplementary Material

## Data Availability

The data that support the findings of this study are available from the corresponding author upon reasonable request.
