# Supplementary material for: Density Functional Theory‐Based Investigation of Adsorption, Permeation, and Desorption of Hydrogen Isotopes in Fe–Cr Alloy of Varied Compositions
Source: Chemphyschem. 2026 Jul 26;27(14):e70501. doi: 10.1002/cphc.70501 (PMC13401912; doi:10.1002/cphc.70501)
Supplement: Supplementary file 1 — Supplementary Material [file CPHC-27-e70501-s001.pdf]

Supplementary Information

Density functional theory based investigation of adsorption, permeation and desorption of hydrogen isotopes in Fe-Cr alloy of varied compositions

Anil Boda[a]*, Sk. Musharaf Ali[a,b]*

[a]Chemical Engineering Division, Bhabha Atomic Research Centre, Mumbai 400085, India

[b]Homi Bhabha National Institute, Mumbai 400094, India

* anilboda@barc.gov.in; musharaf@barc.gov.in

Contents

S1. Variation of Lattice Volume and Density with Cr Content in Bulk Fe

S2. Density Variation with Cr Content

S3. Cohesive energy and bulk modulus of Fe-Cr system

S4. Elastic modulus of Fe-Cr binary system

S5. Effect of vacancy on hydrogen absorption in Fe-Cr (12.5%)

Figure S1. Plot of computed values of lattice volume with variation of Cr content in bulk Fe.

Figure S2. Plot of computed values of Density with variation of Cr content in bulk Fe

Figure S3. Plot of computed values of bulk modulus with variation of Cr content in bulk Fe.

Figure S4. Plot of computed values of cohesive energy with variation of Cr content in bulk Fe.

Figure S5. Optimized bulk structures of six H atoms placed sequentially around vacancy site(Fe) in Fe-Cr.

Table S1: The calculated elastic properties of Fe–Cr binary alloy with experimental reference values

Table S2: Calculated values of absorption energies (eV) of H nearby vacancy sites.

S1. Variation of Lattice Volume and Density with Cr Content in Bulk Fe

The lattice volumes for Fe–Cr solid solutions were calculated with varying Cr content and are presented in **Figure S1**. The results are compared with the ideal behaviour predicted by **Vegard's law of solid solutions,** given as:

$$P = P_A(1-X_B) + P_B\,(X_B) \qquad (1)$$

where **P** is the property (lattice volume or density) of the A–B mixture, $\mathbf{P_A}$ and $\mathbf{P_B}$correspond to the pure components (Fe and Cr), and $\mathbf{X_B}$ is the mole fraction of Cr.

In **Figure S1**, the calculated lattice volumes (DFT) show both **positive and negative deviations** from Vegard's law. Up to approximately **30 at.% Cr**, the Fe–Cr system exhibits a **positive deviation,** indicating a slight expansion of the lattice compared to ideal mixing. Beyond this composition, a **negative deviation** is observed, suggesting contraction relative to the ideal line. These deviations can be attributed to changes in interatomic interactions between Fe–Fe, Cr–Cr, and Fe–Cr pairs. The smaller cohesive energy and bulk modulus of Cr relative to Fe lead to non-linear behaviour in the solid solution. From the experiments, the **lattice constant increases** from approximately **2.87 Å for pure Fe**[1] **to 2.91 Å for pure Cr[1],** corresponding to an **increase of ~1.4 %.** The calculated **lattice volume** increases by about **4.23 %** from pure Fe to pure Cr, consistent with the slightly larger atomic radius of Cr. From the calculated results, the lattice volume increases by 0.67% from pure Fe to Cr due to consideration of 2x2x2 super cell.

S2. Density Variation with Cr Content

The variation of **density** with Cr concentration is shown in **Figure2**. Both DFT-calculated densities and the ideal Vegard's law trend are plotted. The DFT results exhibit a nearly linear decrease in density with increasing Cr concentration, closely following Vegard's prediction, with minor deviations around intermediate compositions (20–40 at.% Cr).Experimentally, the density of Fe is **7.87 g/cm³**[1], and that of Cr is **7.19 g/cm³**[1]corresponding to a **decrease of ~8.64 %.** From the graph, the density decreases from about **8.15 g/cm³ (Fe) to 7.47 g/cm³ (Cr),** corresponding to a **decrease of ~8.34 % which is in good agreement with experimental value**. This reduction is attributed to the higher atomic volume and lower atomic mass density of Cr relative to Fe. The combination of **positive and negative**

deviations in lattice volume and the near-linear decrease in density indicates that Fe–Cr alloys deviate moderately from ideal solid-solution behavior.

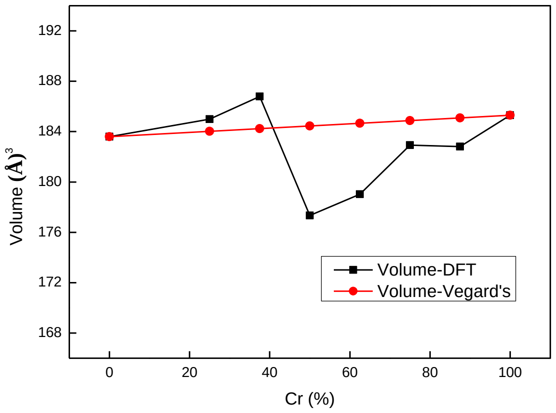

Figure S1. Plot of computed values of lattice volume with variation of Cr content in bulk Fe.

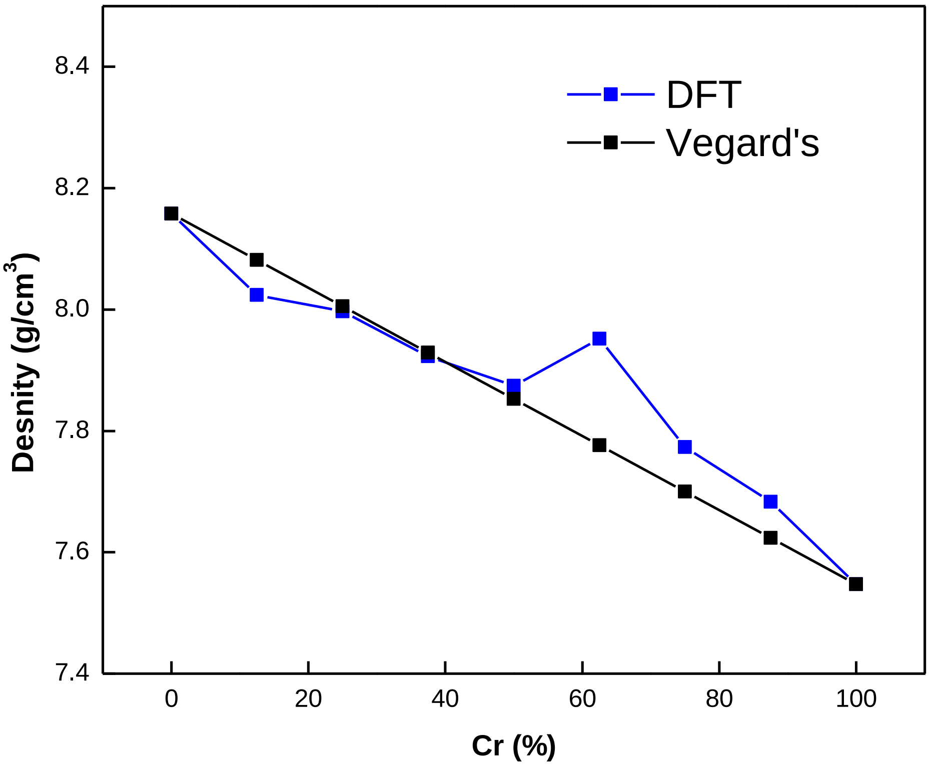

Figure S2.Plot of computed values of Density with variation of Cr content in bulk Fe

S3. Cohesive energy and bulk modulus of Fe-Cr system

The cohesion energy for pure bulk solid is calculated according to the following relation:

$$E_{coh} = E_{at} - E_{bulk}/N \qquad (2)$$

where E_{at} and E_{bulk} is the energy of the isolated atom in vacuum and bulk crystal having N atoms. For Fe-Cr binary system the same may can written as:

$$E_{coh(Fe\text{-}Cr)} = E_{Fe} + E_{Cr} - E_{bulk}/N \qquad (3)$$

The variation of bulk modulus and cohesive energy with Cr concentration, as obtained from the DFT results, is shown in **Figure S3** and **Figure S4**, respectively. From **Figure S3**, the bulk modulus for pure Fe (0% Cr) is approximately 183GPa, while for pure Cr (100% Cr) it increases to about 250 GPa. This represents an increase of around 67GPa, corresponding to **a 36 % rise** in bulk modulus from Fe to Cr. The overall trend shows a non-linear dependence on composition, with the modulus increasing with Cr content and reaching a maximum around the mid-composition range (≈ 60 at.% Cr), indicating enhanced resistance to volume change due to stronger interatomic interactions in mixed Fe–Cr configurations. Experimental bulk modulus for pure Fe is approximately 168 GPa[1], while for pure Cr it is160 GPa[1].

In contrast, the cohesive energy values from **Figure S4** show a slight decrease in magnitude with increasing Cr concentration. The DFT cohesive energy for pure Fe is about −4.99 eV / atom, which becomes −4.05 eV / atom for pure Cr, indicating a reduction of 0.94 eV / atom in magnitude. This suggests that the cohesive binding strength becomes weaker as Cr replaces Fe in the alloy system. The smaller magnitude of cohesive energy for Cr reflects its relatively lower atomic binding strength compared to Fe, consistent with the general trend of reduced metallic bonding strength in Cr-rich compositions. Similar trend is observed in the experimental cohesive energies, i.e. cohesive energy for pure Fe is about −4.28 eV / atom[1], which becomes −4.10 eV / atom for pure Cr[1].

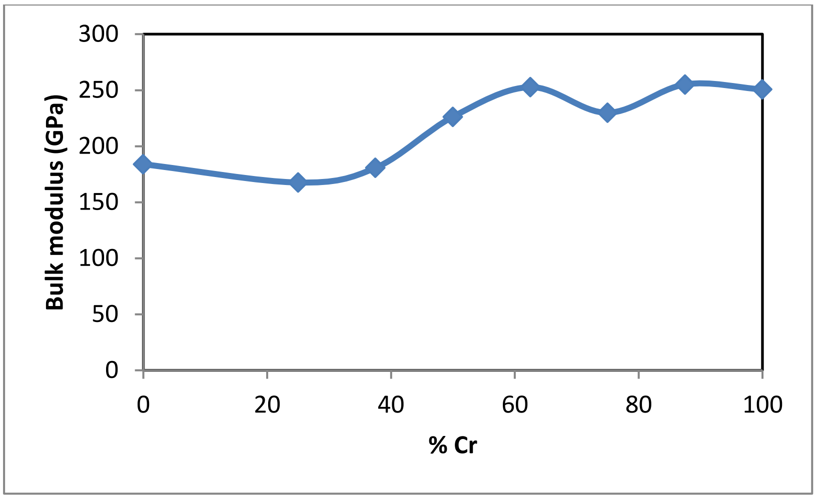

Figure S3. Plot of computed values of bulk modulus with variation of Cr content in bulk Fe.

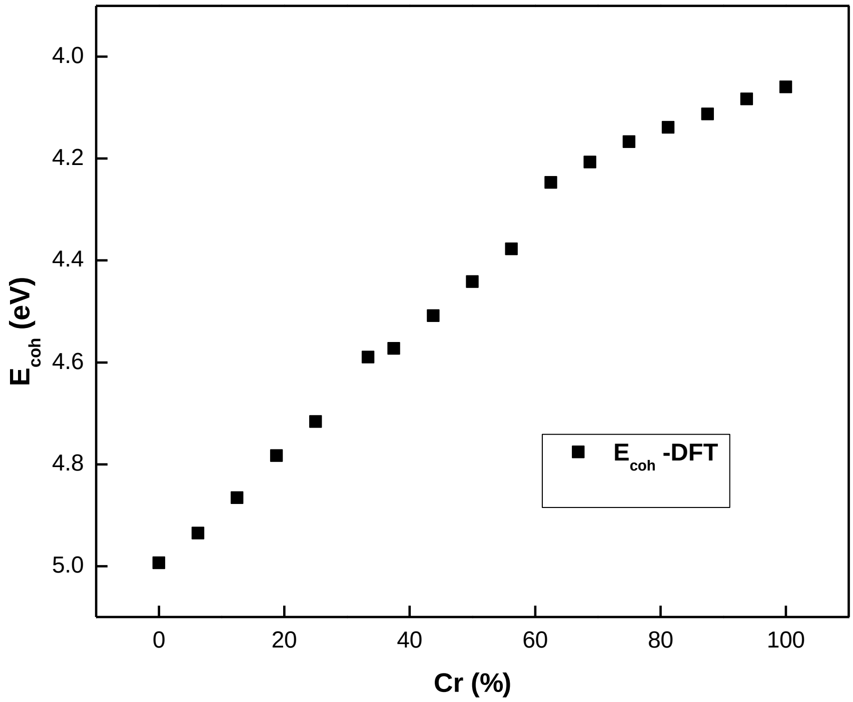

Figure S4. Plot of computed values of cohesive energy with variation of Cr content in bulk Fe.

S4. Elastic modulus of Fe-Cr binary system

Voigt-Reuss-Hill model can be used to calculate Young's modulus (E), bulk modulus (B), shear modulus (G), and Poisson's ratio (ν) of Fe-Cr binary alloy. The modulus obtained from the Voigt model is derived from the Elastic constants C_{ij}, and the modulus obtained from

the Reuss model is derived from the Compliance constants S_{ij}. The subscript Voigt in the following formula represents the Voigt model, and subscript Reuss represents the Reuss model. The *B, G, E* , and ν are calculated as follows, respectively[2, 3]:

$$B_r = \frac{1}{S_{11}+S_{22}+S_{33}+2(S_{12}+S_{23}+S_{13})} \quad (4)$$

$$B_v = \frac{C_{11}+C_{22}+C_{33}+2(C_{12}+C_{23}+C_{13})}{9} \quad (5)$$

$$G_r = \frac{15}{4(S_{11}+S_{22}+S_{33}-S_{12}-S_{23}-S_{13})+3(S_{44}+S_{55}+S_{66})} \quad (6)$$

$$G_v = \frac{C_{11}+C_{22}+C_{33}-C_{12}-C_{23}-C_{13}}{15} + \frac{C_{44}+C_{55}+C_{66}}{5} \quad (7)$$

$$B = \frac{B_r+ B_v}{2} \quad (8)$$

$$G = \frac{G_r+ G_v}{2} \quad (9)$$

$$E = \frac{9BG}{3B+G} \quad (10)$$

$$v = \frac{3B-2G}{2(3B+G)} \quad (11)$$

Table S1: The calculated elastic properties of Fe–Cr binary alloy with experimental reference values

Property	100 % Cr (Computed)	100 % Cr (Exp.)[4]	75 % Cr	50 % Cr	25 % Cr	0 % Cr (Computed)	0 % Cr (Exp.)[5]
C_{11} (GPa)	471.29	350	356.91	306.32	266.60	257.34	242
C_{12} (GPa)	145.54	69, 103	177.65	161.15	137.23	142.36	147
C_{13} (GPa)	145.54	—	177.65	134.61	137.23	142.36	—
C_{22} (GPa)	471.29	—	356.91	306.32	258.80	257.34	—
C_{23} (GPa)	145.54	—	177.65	134.61	131.17	142.36	—
C_{33} (GPa)	471.29	—	356.91	288.92	258.80	257.34	—
C_{44} (GPa)	85.25	101	99.83	108.81	115.31	112.15	112
C_{55} (GPa)	85.25	—	99.83	45.81	113.61	112.15	—
C_{66} (GPa)	85.25	—	99.83	45.81	115.31	112.15	—
Voigt Bulk Modulus (Bv) (GPa)	254.13	162, 198	237.40	195.81	177.27	180.68	166, 175

Reuss Bulk Modulus (Br) (GPa)	254.13	—	237.40	194.96	177.15	180.68	—
VRH Bulk Modulus (B) (GPa)	254.13	—	237.40	195.39	177.21	180.68	—
Voigt Shear Modulus (Gv) (GPa)	116.30	—	95.75	71.50	94.09	90.29	
Reuss Shear Modulus (Gr)(GPa)	105.33	—	95.49	63.68	86.42	81.25	—
VRH Shear Modulus (G)(GPa)	110.81	—	95.62	67.59	90.25	85.77	—
Young's Modulus (GPa) (E)	290.25		252.90	181.81	231.46	222.15	
Poisson's Ratio (μ)	0.310	0.29	0.322	0.345	0.282	0.295	0.29

The Table 1 presents the computed and experimental elastic properties of Fe–Cr binary alloys across different chromium concentrations, ranging from pure Fe (0% Cr) to pure Cr (100% Cr). The calculated values of the single-crystal elastic constants (Cij), bulk modulus (B), shear modulus (G), Young's modulus (E), and Poisson's ratio (μ) are listed and compared with available experimental data for pure Fe and Cr. For pure bcc Cr, the computed elastic constants C_{11}, C_{12}, and C_{44} are 471.29 GPa, 145.54 GPa, and 85.25 GPa, respectively, which are higher than the experimental values of 350 GPa, 69–103 GPa, and 101 GPa, indicating a slightly stiffer lattice in the computed model. Similarly, for pure bcc Fe, the computed values C_{11} = 257.34 GPa, C_{12} = 142.36 GPa, and C_{44} = 112.15 GPa are in reasonable agreement with experimental data (C_{11} = 242 GPa, C_{12} = 147 GPa, C_{44} = 112 GPa) [5].

As the chromium concentration increases from 0% to 100%, the elastic constants generally increase, reflecting the higher stiffness of Cr compared to Fe. The bulk modulus (VRH average) increases from 180.68 GPa in pure Fe to 254.13 GPa in pure Cr, while the shear

modulus rises from 85.77 GPa to 110.81 GPa, showing a clear compositional dependence. Correspondingly, Young's modulus (E) follows a similar trend, ranging from 222.15 GPa (pure Fe) to 290.25 GPa (pure Cr). The Poisson's ratio slightly decreases from 0.345 (at 50% Cr) to 0.282 (at 25% Cr) and finally to 0.310 for pure Cr, indicating moderate changes in elastic anisotropy and ductility with alloy composition. Overall, the computed results show good consistency with experimental data and demonstrate a systematic variation of mechanical properties with chromium concentration in the Fe–Cr alloy system.

S5. Effect of vacancy on hydrogen absorption in Fe-Cr (12.5%)

In order to examine the effect of vacancy, we have removed one Cr to form a Cr-vacancy in bulk Fe-Cr super cell containing 128 atoms**.** Similarly,one Fe removed to form a Fe-vacancy in bulk Fe-Cr super cell containing 128 atoms. Further, sequentially the H atoms were placed near to vacancy in the octahedral position. There are six octahedral positions near the vacancy.

The hydrogen-vacancy binding energy is defined as,

$$E_{(VHn+H)} = E_{(FeCr127VHn+1)} + E_{(FeCr128)} - E_{(FeCr127VHn)} - E_{(FeCr128H)} \qquad (12)$$

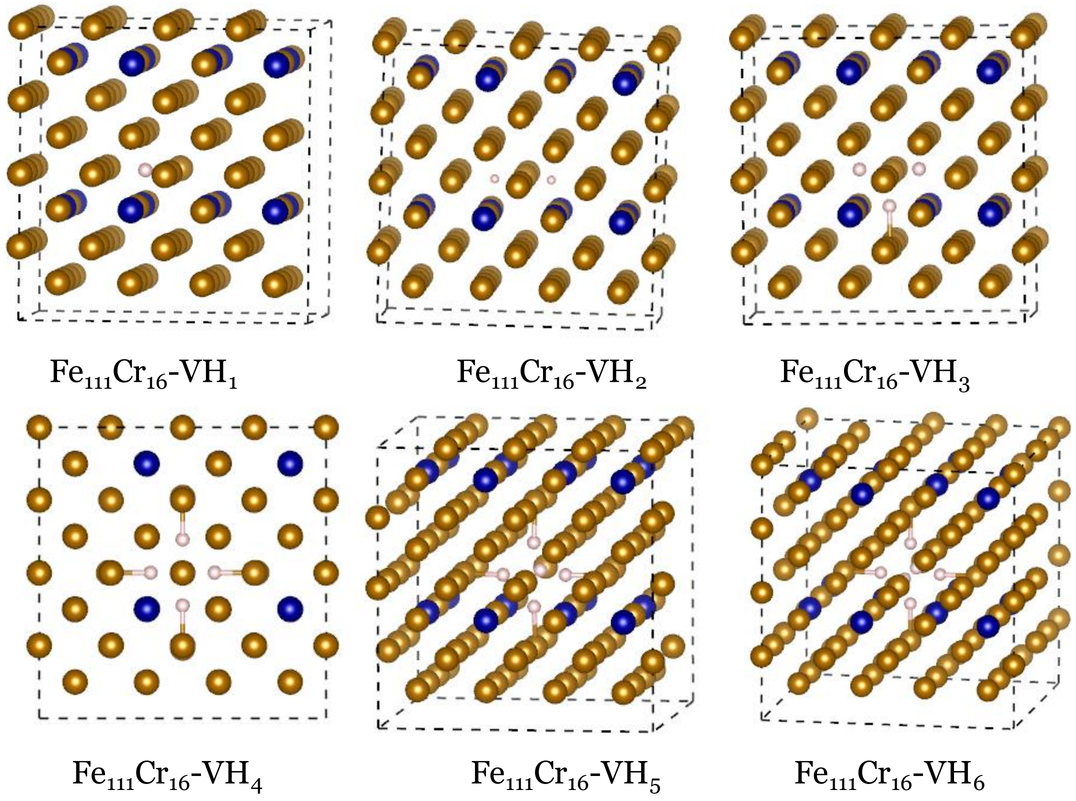

Figure S5. Optimized bulk structures of six H atoms placed sequentially around vacancy site(Fe) in Fe-Cr.

The vacancy-binding energy calculations indicate that hydrogen binding to vacancies is exothermic up to six H atoms in the case of a Cr vacancy, whereas only up to two H atoms can stably bind to an Fe vacancy. As the number of hydrogen atoms increases, the hydrogen-vacancy binding energy decreases, suggesting a weakening interaction due to increased hydrogen repulsion or saturation effects. Additionally, the binding energy for T (tritium) in vacancies is found to be lower compared to D (deuterium) and H (protium), reflecting isotope-dependent differences in vacancy interactions, likely influenced by zero-point energy contributions.

Table S2: Calculated values of absorption energies (eV) of H nearby vacancy sites.

	**Cr-Vacancy**			**Fe-vacancy**		
	**H**	**D**	**T**	**H**	**D**	**T**
VH	-0.106	-0.074	-0.059	-0.033	-0.005	0.008
VH_2	-1.454	-1.423	-1.409	-0.076	-0.050	-0.038
VH_3	-0.497	-0.486	-0.481	0.198	0.213	0.220
VH_4	-0.574	-0.515	-0.489	0.136	0.162	0.174
VH_5	-0.387	-0.422	-0.437	0.929	0.931	0.932
VH_6	-0.468	-0.390	-0.356	0.083	0.125	0.143

References

[S1] C. Kittel *John Wiley & Sons, New York* **1996**.
[S2] Q.-J. Liu, Z.-T. Liu *Materials science in semiconductor processing*. **2014**, 27, 765-776.
[S3] Q. Song, Y.-Q. Hou, L.-S. Li, Z.-Y. Jiang, B. Zhou, X.-D. Zhang *Physica B: Condensed Matter*. **2012**, 407, 565-570.
[S4] V. I. Razumovskiy, A. V. Ruban, P. A. Korzhavyi *Physical Review B—Condensed Matter and Materials Physics*. **2011**, 84, 024106.
[S5] K. Masuda, N. Hamada, K. Terakura *Journal of Physics F: Metal Physics*. **1984**, 14, 47.
